# *Pandoraea* sp. RB-44, A Novel Quorum Sensing Soil Bacterium

**DOI:** 10.3390/s131014121

**Published:** 2013-10-18

**Authors:** Robson Ee Han-Jen, Yin Wai-Fong, Chan Kok-Gan

**Affiliations:** Division of Genetics and Molecular Biology, Institute of Biological Sciences, Faculty of Science, University of Malaya, 50603 Kuala Lumpur, Malaysia; E-Mails: robsonee@live.com (R.E.H.-J.); yinwaifong@yahoo.com (Y.W.-F.)

**Keywords:** *Pandoraea* sp., matrix-assisted laser desorption ionization time-of-flight (MALDI-TOF), mass spectrometry, triple quodruopole liquid chromatography mass spectrometry, *N*-octanoylhomoserine lactone (C8-HSL), quorum sensing

## Abstract

*Proteobacteria* are known to communicate *via* signaling molecules and this process is known as quorum sensing. The most commonly studied quorum sensing molecules are *N*-acylhomoserine lactones (AHLs) that consists of a homoserine lactone moiety and an *N*-acyl side chain with various chain lengths and degrees of saturation at the C-3 position. We have isolated a bacterium, RB-44, from a site which was formally a landfill dumping ground. Using matrix-assisted laser desorption ionization time-of-flight (MALDI-TOF) mass spectrometry analysis, this isolate was identified as a *Pandoraea* sp.which was then screened for AHL production using biosensors which indicated its quorum sensing properties. To identify the AHL profile of *Pandoraea* sp. RB-44, we used high resolution tandem mass spectrometry confirming that this isolate produced *N*-octanoylhomoserine lactone (C8-HSL). To the best of our knowledge, this is the first report that showed quorum sensing activity exhibited by *Pandoraea* sp. Our data add *Pandoraea* sp. to the growing number of bacteria that possess QS systems.

## 1. Introduction

Initially often reported as non-fermentative Gram-negative bacilli or misidentified as *Burkholderia cepacia* complex (Bcc) or *Ralstonia* spp. in clinical microbiology laboratory [1–3], *Pandoraea* sp. is actually a taxonomically distinct β-subclass of proteobacteria where genera of *Bukholderia* and *Ralstonia* are the closest neighbors [4]. As an opportunistic pathogen with an unclear role in clinical colonization, *Pandoraea* spp. have been isolated from various patients, mainly with cystic fibrosis [5–8]. *Pandoraea* spp. are also well known for their bio-catalytic activities in a variety of reactions such as carbapenem hydrolysis [9] polychlorinated biphenyls (PCBs) biodegradation [10], sulphur oxidation [11] and lignin degradation [12]. Despite all the documentation on this species, little is known about the mechanism and regulation of its pathogenicity and bio-catalytic activities.

Most Gram negative bacteria use quorum sensing (QS) to establish communications between neighboring cells through secretion and detection of diffusible signaling molecule known as autoinducers [13–15]. QS is important as it regulates diverse bacterial physiological activities, such as swimming and twitching motility, bioluminescence, swarming, stimulate production of virulence in flora and fauna pathogens, biofilm differentiation, antibiotic biosynthesis, plasmid conjugal transfer and many more activities [13,16,17]. By far, the most widely studied QS molecules are *N*-acyl-homoserine lactones (AHLs) which are synthesized by AHL synthase (*luxI* homologue) that will diffuse and bind to its cognate receptor (*luxR* homologue) where this AHL-LuxR complex will be activated to regulate QS-mediated genes expression [18,19]. Thus, the best way to understand the molecular basis of *Pandoraea* sp. virulence factor and bio-catalytic activities is through characterizing its AHL production expression [20].

Here, we present a strain of *Pandoraea* sp. RB-44 isolated through four cycles of enrichment in KGm medium [21] from an ex-landfill site that activates both *C. violaeum* CV026 and *E. coli* [pSB401] biosensors. Subsequently, we used matrix-assisted laser desorption ionization time-of-flight (MALDI-TOF) mass spectrometry analysis for strain identification and high resolution quadrupole mass spectrometry to confirm its AHL profile. To our knowledge, this is the first report of AHL production by *Pandoraea* sp.

## 2. Experimental Section

### 2.1. Soil Sampling

Soil sampling was conducted in 2012 from an ex-landfill site in Ayer Hitam, Puchong (Malaysia) with the GPS coordinates of N03′00′12.1, E101′39′33′1 at an elevation of 61 m above sea level. The soil sample was collected from the soil surface at a depth of 10 cm and soil sample was placed into a sterile 50mL plastic tube. Upon arrival in the laboratory, the soil sample was processed with removal of large particulate organic matter using a sterile spatula.

### 2.2. Enrichment and Isolation

Enrichment and isolation of bacteria was done according to a previously described method [22] with slight modifications. Briefly, a soil sample (1 g) was resuspended in KGm medium (10 mL). The mixture was vigorously vortexed and the soil suspension was transferred (10% v/v) into fresh KGm medium supplemented with 3-oxo-C6-HSL (50 mM final concentration, Sigma-Aldrich, St Loius, MO, USA) as the sole carbon sources. The mixture was incubated in 28 °C with shaking (220 rpm) for 48 h. Similar transfers were repeated three times. At the fourth enrichment cycle, a diluted suspension was plated on Luria-Bertani (LB) agar and a plate of 3-oxo-C6-HSL-containing KGm agar to isolate pure colonies.

### 2.3. Bacteria Strains and Culture Conditions

All strains (Table 1) were routinely grown aerobically in LB medium with shaking (220 rpm) and LB agar at 28 °C unless otherwise stated. The composition of LB medium was: tryptone (10 g/L), yeast extract (5 g/L) and sodium chloride (10 g/L) with additional bacto-agar (15 g/L) in LB agar. For AHL extraction, bacteria strains were grown in a modified LBmedium buffered with 50 mM 3-[*N*-morpholino] propanesulfonic acid (MOPS) to pH 5.5 to prevent alkaline hydrolysis of any AHLs.

### 2.4. Detection of AHL Production Using C. Violaeum CV026 Biosensor

Preliminary screening of AHL production was done by cross streaking the isolate with *C. violaeum* CV026 on LB agar and incubated for 24 h at 28 °C. Fomation of purple pigmentation after 24 h of incubation indicates production of short chain exogenous AHLs from the isolate. *E. carotovora* GS101 and *E. carotovora* PNP22 was used as positive and negative controls, respectively.

### 2.5. AHL Extraction

Pure culture of isolate RB-44 was grown in LB broth buffered to pH 5.5 with 50 mM MOPS and incubated at 28 °C with shaking for 18 h. The spent supernatant was extracted twice with equal volume of acidified (0.1% v/v glacial acetic acid) ethyl acetate as described preciously [25]. AHL extracts was desiccated to complete dryness before further analysis.

### 2.6. Bioluminescence Assay Using E. coli [pSB401]

Bioluminescence expression was determined using Infinite M200 luminometer (Tecan, Männerdorf, Switzerland) in a 96-well microtitre plates as described previously with slight modification [26]. Briefly, overnight culture of biosensor (*E. coli* [pSB401]) was diluted to an OD_600nm_ of 0.1. Then, diluted biosensor cells culture (250 μL) was used to resuspend the extracted AHL and added into each well of 96-well microtitre plates. Bioluminescence and optical density (OD_495nm_) were determined at 60 min intervals for 24 h. Bioluminescence expression was expressed as relative light unit per OD_495nm_ (RLU/OD_495nm_) against time [26]. Experiments were conducted in triplicate and repeated thrice.

### 2.7. Strain Identification Using 16S rDNA Amplification

The 16S rDNA was PCR amplified using 27F forward primers (5′-AGAGTTTGATCMTGGCTCAG-3′), 515F forward primers (5′–GTGCCAGCMGCCGCGGTAA-3′) and 1525R reverse primers (5′–AAGGAGGTGWTCCARCC-3′) using PCR mix (Promega Kit, Madison, WI, USA) while the genomic DNA was extracted using MasterPure™ DNA Purification Kit (EPICENTRE Inc., Madison, WI, USA). The PCR amplification was carried out consisting of an initial denaturation of 94 °C for 3 min, followed by 30 repeated cycles of 94 °C for 30 s of denaturing, 60 °C for 30 s of annealing and 72 °C for 1 min 30 s of extension, and a final extension of 72 °C for 7 min. Product sequence alignment was done using GenBank Blastn database and phylogenetic analysis was done using Molecular Evolutionary Genetic Analysis (MEGA) version 5.0 [27].

### 2.8. Matrix-Assisted Laser Desorption Ionization Time-of-Flight Mass Spectrometry (MALDI-TOF MS) Identification

Sample preparation for MALDI-TOF MS analysis was conducted as described previously [28]. Briefly, fresh culture on LB agar was smeared on MSP 96 target polished steel BC plate and overlaid with 1 μL of MALDI matrix (10 mg/mL α-cyano-4-hydroxycinnamic acid in 50% acetonitrile/2.5% trifluoroacetic acid). The sample was air dried before further analysis in Microflex MALDI-TOF (Bruker Daltonik GmbH, Leipzig, Germany) bench-top mass spectrometer (equipped with UV laser at wavelength 337 nm). The built in support software used was Bruker FlexControl software version 3.3 (Build 108) and the spectra were recorded in the linear positive ion mode and analyzed the mass in range of 2 to 20 kDa. Each well on the sample plate was measured with laser shots at various random positions and the bacterial MS spectra was analyzed using Bruker MALDI Biotyper Real Time Classification Classification software (Version 3.1, Build 65). The identity of the sample was evaluated based on a dedicated scoring system where the spectra information of the sample was compared to the best match in the Bruker database. If the final score of the spectra value is between 2.3 to 3.0, the accuracy of the isolate identity is accurate until the level of species; for spectra values between 2.3 to 3.0, the accuracy is secure at the level of genus; for spectra values between 1.7 to 2.0, the accuracy of identity is below the level of genus and for values lower than 1.7, no reliable identity is generated. A dendrogram was then constructed from the standard MALDI Biotyper MSP creation method (Bruker Daltonics, Bremen, Germany) where a phylogenetic tree was generated by similarity scoring of a set of mass spectra illustrating graphical distance values between species constructed from their MALDI-TOF reference spectra.

### 2.9. Identification of AHL Profile by High Resolution Tandem Liquid Chromatography Quadrupole Mass Spectrometry (LC-MS/MS)

An Agilent 1290 Infinity LC system (Agilent Technologies Inc., Santa Clara, CA, USA) was used as the LC delivery system. The column used was the HPLC column C18 column (Agilent Technologies Inc., Santa Clara, CA, USA); 2.1 mm × 50 mm, 1.8 μm particle size where the injection volume was set to 2 μL. LC analysis was carried out at 37 °C column temperature with an elution of 15 min at constant flow rate of 0.3 mL/min. Mobile phase A and B were 0.1% v/v formic acid in HPLC grade MilliQ water and 0.1 % v/v formic acid in acetonitrile (ACN) respectively. The gradient profiles for the HPLC condition were as follows at (time: mobile phase A: mobile phase B): 0 min: 80:20, 7 min: 50:50, 12 min: 20:80, and 14 min: 80:20.

High Resolution Tandem LC-MS/MS was performed with the Agilent 6490 Triple Quadrupole LC/MS system (Agilent Technologies Inc., Santa Clara, CA, USA). The ion source used is in electrospray ionization (ESI) positive mode.The probe capillary voltage was set at 3 kV, nebulizer pressure at 20 psi, sheath gas at 11 mL/h, desolvation temperature at 200 °C, collision energy at 5 eV and fragmentation at 380 eV. For detection of AHLs, precursor ion scan mode is used where the product ion *m/z* was set as 102 indicating the [M+H]^+^ ion of the core lactone ring moiety. The *m/z* value range of the precursor ions was then set from 150 to 400. Agilent Mass Hunter software was used for the MS data analysis by comparison of extracted ion (EI) mass spectra and retention index with synthetic AHL compounds.

## 3. Results and Discussion

### 3.1. Sampling and Screening for AHL Producing Bacteria

The sample site was previously a landfill site for domestic household waste materials and had ceased operation since 7 years ago. The temperature of the soil surface was 33 °C in daytime and pH reading of value 7 was documented on the day when sampling was done. The soil sample was collected in the middle of the ex-landfill site.

A wide variety of bacterial biosensors have been constructed for screening of AHL production [23,24,29]. Most of these biosensors function with a mutated AHL synthase but with a functional AHL receptor remains intact that could respond to the presence of exogenous AHLs by the activation of a reporter gene such as *lacZ* or *lux operon*. In this study, *C. violaceum* CV026 biosensor was used for routine screening as it is a rapid and accurate preliminary QS screening [23]. Subsequently, further analysis was conducted with bioluminescence assay in *lux*-based *E. coli* biosensors followed by high resolution analytical instrument such as mass spectrometry analysis.

**Figure 1.**
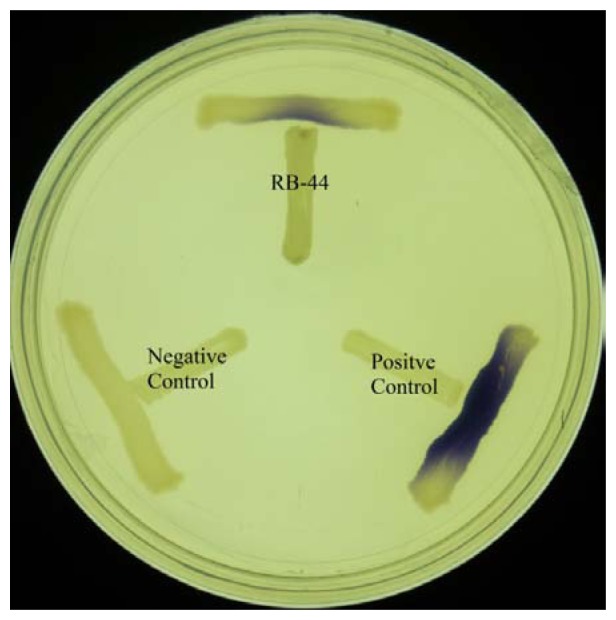
AHL screening of strain RB-44 with *C. violaeum* CV026. *E. carotovora* GS101 and *E. carotovora* PNP22 refer to the postive and negative controls, respectively.

**Figure 2.**
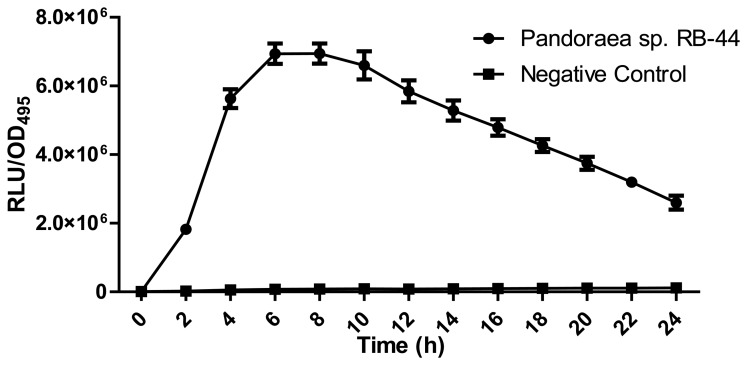
Bioluminescence assay using *E. coli* [pSB401] biosensor. Graph was plotted as RLU/OD_495nm_ against time. AHL production by strain RB-44 (circle) is confirmed the increased value of RLU/OD_495nm_ and observed over 24 h. Negative control (square) was included with extracted AHL in blank LB. Each point represents the mean and error of results from independent triplicate cultures.

About 60 strains were isolated after four cycles of enrichment in KGm medium with 3-oxo-C6-HSL as the sole carbon sources. Preliminary screening for AHL production using *C. violaeum* CV026 biosensor (Figure 1) suggested strain RB-44 showed QS activity and moderate quorum quenching actitivity for 3-oxo-C6-HSL (data not shown). Strain RB-44 was selected for further QS analysis since it triggered *C. violaeum* CV026 biosensor purple pigement formation and subsequently reconfirmed its AHL production by using *E. coli* [pSB401] bioluminescence assay (Figure 2).

### 3.2. Strain Identification and MALDI-TOF Score-Oriented Dendrogram

There are many reports in clinical laboratories that conventional methods are not able to identify *Pandoraea* spp. even when using a widely accepted phenotypic method [30,31]. There is even a quality assurance trial of CF microbiology which *Pandoraea pnomenusa* strain was misclassified or not even detected by many laboratories that reinforced this notion [32]. Thus, MALDI-TOF mass spectrometry represents a more reliable method and high throughput for the classification and identification of microorganisms with a shorter turn over time [33,34]. Unlike other cystic fibrosis-causing agents, no biofilm formation is observed from overnight culture of strain RB-44.

**Figure 3.**
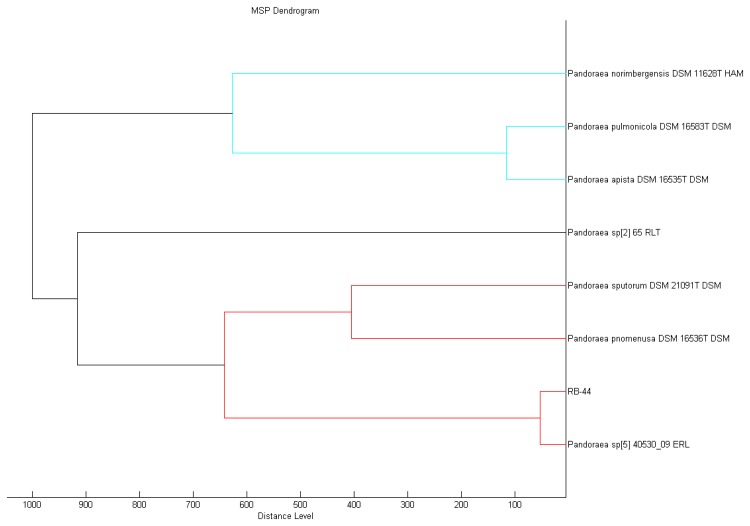
Score-orientated dendrogram that shows classification of *Pandoraea* sp. RB-44. Bacterial strain RB-44 is clustered hierarchically based on the protein mass spectra patterns.

MALDI-TOF MS is a rapid and yet promising means of accurate identification of microorganisms [35], especially for the *Pandoraea* genus that is often misidentified as *Burkholderia* spp. or *Ralstonia* spp. [31]. In this study, MALDI-TOF MS result accurately showed that the closest match of the RB-44 isolate is *Pandoraea* sp.with a best match score value of 2.415 and *P. pnomenusa* as the second best match (score value of 2.382) in the MALDI-TOF Biotyper Real Time Classification software. MALDI Biotyper MSP software also showed a closer match to *Pandoraea* sp. (Figure 3).

**Figure 4.**
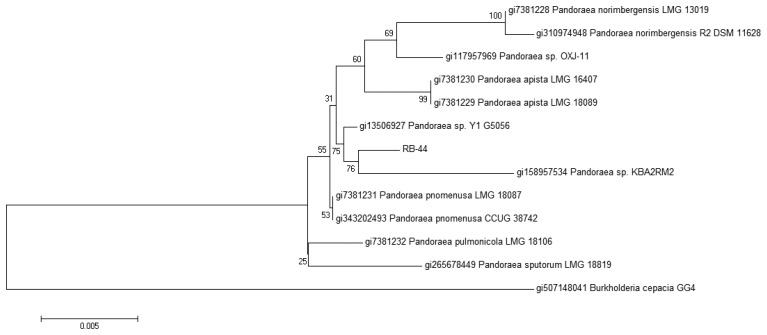
The evolutionary history was inferred using the Neighbor-Joining method [36]. The optimal tree with the sum of branch length = 0.08118917 is shown. The percentage of replicate trees in which the associated taxa clustered together in the bootstrap test (1,000 replicates) are shown next to the branches [37]. The tree is drawn to scale, with branch lengths in the same units as those of the evolutionary distances used to infer the phylogenetic tree. The evolutionary distances were computed using the Maximum Composite Likelihood method [38] and are in the units of the number of base substitutions per site. The analysis involved 13 nucleotide sequences. Codon positions included were 1st+2nd+3rd+Noncoding. All positions containing gaps and missing data were eliminated. There were a total of 1388 positions in the final dataset. Evolutionary analyses were conducted in MEGA 5.0 [27].

The identity of strain RB-44 is also confirmed by 16S rDNA sequencing showing that it clusters within the Pandoraea genus. The sequence was subsequently deposited into GenBank with the accession number KF648559. A phylogenetic tree was then constructed using Mega 5 software to align it with other rDNA sequences obtained from GenBank. Based on the phylogenetic tree obtained from MALDI-TOF and 16S rDNA sequencing (Figure 4), it appears that strain RB-44 could represent a new species of *Pandoraea* genus. However, more research should be to be conducted to confirm this finding.

### 3.3. Identification of AHL Profile by High Resolution Tandem Liquid Chromatography Quadrupole Mass Spectrometry (MS/MS)

**Figure 5.**
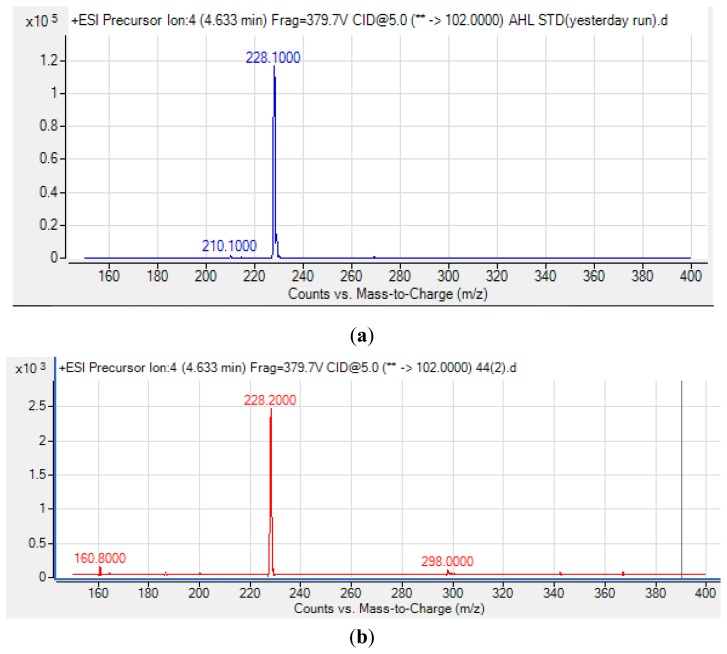
Mass spectrometry analysis of C8-HSL from an AHL standard (**a**) showed the *m/z* value 228.100; retention time: 4.633 min; abundance: 115,382.02 and abundance %: 100, while C8-HSL produced by *Pandoraea* sp. RB-44 (**b**) showed *m/z* value: 228.200; retention time: 4.633 min; abundance: 2,459.14 and abundance %: 100.

Many cystic fibrosis-causing agents such as *Pseudomonas aeruginosa* and *Burkholderia* spp. have been reported to possess QS properties as a mechanism to regulate virulence. As an opportunistic pathogen with an unclear role, *Pandoraea* spp. have repeatedly been isolated and reported to be co-colonizing species in cystic fibrosis patients [5,31,34]. Thus, identification of the *Pandoraea* spp. AHL profile is the first step to understand this potential opportunistic pathogen. The spent culture supernatants of *Pandoraea* sp. RB-44 was analyzed using an Agilent 6490 Triple Quadrupole LC-MS/MS system. Mass spectrometry analysis of the supernatant of *Pandoraea* sp. RB-44 confirmed the presence of *N*-octanoylhomoserine lactone (C8-HSL) (*m/z* value of 228.200) (Figure 5).

## 4. Conclusions/Outlook

We report here the AHL profile of *Pandoraea* sp. RB-44 isolated from an ex-landfill site. A short chain AHL, namely C8-HSL, was detected in the spent culture supernatant of *Pandoraea* sp. RB-44. To the best of our knowledge, this is the first documentation of AHL production by a member of the *Pandoraea*. Further investigations are being carried out to confirm the QS gene of *Pandoraea* sp. RB-44.

## Figures and Tables

**Table 1.** Strains used in this study.

**Strain**	**Description**	**Source/Reference**
*Chromobacterium violaceum* CV026	Double mini-Tn*5* mutant derived from ATCC31532. Biosensor that synthesize purple violacein pigment in response of short chain exogenous AHL.	[23]
*Erwinia carotovora* GS101	Positive control for QS properties. Capable of producing AHL to activate *C. violaeum* CV026.	Gift from Prof. Paul Williams
*Erwinia carotovora* PNP22	Negative control for QS properties.	Gift from Prof. Paul Williams
Escherichia coli [pSB401]	*luxRluxl'* (*Photobacterium fischeri* [ATCC 7744]):*luxCDABE* (*Photorhabdus luminescens* [ATCC 29999]) fusion; pACYC184-derived, *tet^R^*. Biosensor that produces bioluminescence in the presence of short chain exogenous AHL.	[24]
*Pandoraea* sp. RB-44	Soil isolate	This study
